# Structuring of Surface Films Formed on Magnesium in Hot Chlorobenzotriazole Vapors

**DOI:** 10.3390/ma15196625

**Published:** 2022-09-23

**Authors:** Olga A. Goncharova, Andrey Yu Luchkin, Ivan N. Senchikhin, Yury B. Makarychev, Victoriya A. Luchkina, Olga V. Dement’eva, Sergey S. Vesely, Nickolay N. Andreev

**Affiliations:** A.N. Frumkin Institute of Physical Chemistry and Electrochemistry, Russian Academy of Sciences, Leninskii pr. 31, 119071 Moscow, Russia

**Keywords:** magnesium alloy, EIS, scanning electron microscopy, atmospheric corrosion, structuring of surface films, passivity, corrosion inhibitors

## Abstract

Chamberprotection of metals from atmospheric corrosion is a variety of vapor-phase inhibition. It is based on the effect of adsorption films formed in the vapors of low-volatile corrosion inhibitors at elevated temperatures. The paper analyzes the specific features of the chamber protection of a magnesium alloy with chlorobenzotriazole. It has been found that the protective properties of surface films formed in hot vapors of this compound increase upon exposure of the metal to air. The processes of structuring of protective films that occur in this case have been studied by a set of corrosion, electrochemical and physical methods. It has been shown that chamber treatment of the alloy is accompanied by chlorobenzotriazole adsorption and uniform thickening of the surface oxide-hydroxide layer. In this case, the corrosion processes slow down by a factor of up to 10. Prolonged exposure of the samples in air after the chamber treatment results in additional oxidation of magnesium and hydroxylation of the oxide. However, the oxide-hydroxide layer does not grow on the entire surface, but as separate islets. Such a change in the structure of the surface films results in an additional 10-fold increase in the corrosion resistance of the magnesium alloy.

## 1. Introduction

Due to unique physical and mechanical properties of magnesium, its alloys are promising structural materials [1,2]. However, they readily undergo corrosion under weathering conditions [3,4,5,6]. This can result in a loss of mechanical strength and decorative properties of products, as well as their failure [7].

The use of corrosion inhibitors is a quickly growing field in the protection of magnesium and its alloys [8,9,10,11,12,13,14,15,16,17,18]. Corrosion inhibitors can be used alone [19,20,21,22,23] or as additives that enhance the anticorrosive properties of paint coatings, conversion coatings, and other types of coatings [24,25,26,27,28].

The action of inhibitors is based on the formation of surface films on the metal being protected [29,30]. Such films can affect the kinetics of electrode processes and/or mechanically block the surface from corrosive agents [31]. Many works on magnesium corrosion inhibitors have been published in the past few decades [8,9,10,11,12,13,14,15,16,17,18,19,20,21,22,23,24,25,26,27,28]. As a rule, inhibitors are applied to a surface from solutions. Currently, there are few works on the vapor-phase protection of magnesium and its alloys by inhibitors [32,33].

Vapor-phase protection of metals usually implies the use of volatile inhibitors. However, the so-called “chamber” protection is being intensely developed at present [34,35,36,37,38,39,40,41,42]. It involves a short-term surface treatment with vapors of an organic inhibitor at elevated temperatures. Provided that the reagents and conditions are chosen correctly, this “chamber” treatment (CT) creates protective nanoscale layers with a pronounced anti-corrosion aftereffect on the metal surface.

Compared to the traditional methods of inhibitory protection, the “chamber” passivation has a number of competitive advantages, which are as follows:

- The consumption of an inhibitor is very small;
- There is no waste to be disposed of;
- It is efficient for items with complex geometry ^1^;
- Its safety and environmental friendliness ^2^.

We have shown [32] that 5-chloro-1,2,3-1H-benzotriazole (CBTA) films obtained by 1-h CT at 150 °C can greatly increase the corrosion resistance of a magnesium alloy. A specific feature of such films is that they are capable of structuring upon exposure to the air. Their protective effect increases for some time after the CT. The purpose of this work is to study the structurization processes in surface films that occur during exposure of magnesium samples in the air after CT in CBTA vapor and result in an increase in the corrosion resistance of the metal.

^1^ Including those with blind holes, slots and gaps.

^2^ Chamber inhibitors are present only in the working chamber and are only active at elevated temperatures.

## 2. Experimental

### 2.1. Reagents and Materials

All the reagents used in this study were of “pure” grade. Samples and electrodes made of MA8 magnesium alloy were used in the studies. The composition of the alloy is presented in Table 1.

### 2.2. Samples and Electrodes

We used flat specimens with holes for mounting in the chambers and test cells. The specimen size was 30 × 50 × 4 mm in the case of corrosion tests and 8 × 8 × 2 mm in the X-ray photoelectron spectroscopy and electron microscopy studies.

Cylindrical electrodes with a threaded joint on one of the butt-ends were used in the electrochemical experiments. The electrodes were mounted with an epoxy binder to prevent exposure of their side surfaces to the electrolyte during the experiment. The lower cylinder butt-end with a surface of 0.5 cm^2^ served as the working surface.

Before the thermal treatment (TT) or CT, the samples and electrodes were polished to a mirror finish with sandpaper of various grit sizes, degreased with acetone, and dried. The prepared samples and electrodes were mounted in sealed 0.5 L glass cells, each containing a weighted portion (0.5 g) of CBTA. The cells were tightly sealed and placed for 1 h in a drying cabinet heated to 150 °C ^3^. Air was not removed from the cells. After that, the cells were removed from the cabinet. The samples were withdrawn, kept for a certain time (*τ*_exp_) at room temperature in the air, and tested.

To analyze the processes that occur in the surface films upon exposure of the samples under ambient conditions by electrochemical and spectral methods, the following basic metal preparation conditions were used:

- Magnesium alloy not subjected to TT or CT and exposed for 1 h in air after polishing and degreasing (preparation mode **A**);
- Magnesium alloy after 1-h TT at 150 °C. Exposure time in air after TT—48 h (preparation mode **B**);
- Magnesium alloy after 1-h CT at 150 °C. Exposure time in air after CT—48 h (preparation mode **C**);
- Magnesium alloy after 1-h TT at 150 °C. Time of exposure in air after TT—432 h (preparation mode **D**);
- Magnesium alloy after 1-h CT at 150 °C. Time of exposure in air after TT—432 h (preparation mode **E**).

Apart from the metal preparation conditions described above, corrosion tests were performed using samples with *τ*_exp_ from 1 to 432 h, while scanning electron microscopy was performed with samples with *τ*_exp_ of 24 and 720 h.

^3^ According to a previous publication [32], these are the optimal conditions for the chamber protection of magnesium with CBTA.

### 2.3. Corrosion Tests

The protective aftereffect of films formed during CT was estimated at 100% humidity with recurrent condensation of moisture on the samples. The samples were mounted on nylon threads on the lids of sealed glass cells so that they did not contact each other and the cell walls. The working volume of a cell was 0.6 L. At the beginning of the experiment, 0.1 L of water was poured onto the bottom of each cell. The cells were placed in a heating cabinet preheated to 40 °C and this temperature was maintained for 8 h. After that, heating was turned off and the cabinet was left to cool for 16 h. The heating-cooling cycle was repeated many times. The samples were inspected once an hour without opening the cells. During the tests, the time until corrosion damage appeared on the alloy (*τ*_prot_) was determined.

### 2.4. Voltammetry

Potentiodynamic experiments were performed using an IPC-pro potentiostat (Russian Federation) and a standard three-electrode cell with divided electrode spaces. A platinum wire was used as the auxiliary electrode. The potentials (*E*) were measured against a saturated silver/silver chloride reference electrode and then converted to the standard hydrogen scale. The experiments were performed in 0.05 M NaCl solution. The electrodes were placed in a cell with an electrolyte, kept for 5 min until the potential stabilized (*E*_st_), and polarized anodically from this potential. The potential sweep rate was 0.2 mV/s. The following characteristic values of the anodic polarization curves were used: *E*_st_ and the *E* values (*E_i_*) corresponding to a current density (*i*) of 100 μA/cm^2^.

### 2.5. Electrochemical Impedance Spectroscopy

**Figure 1:**
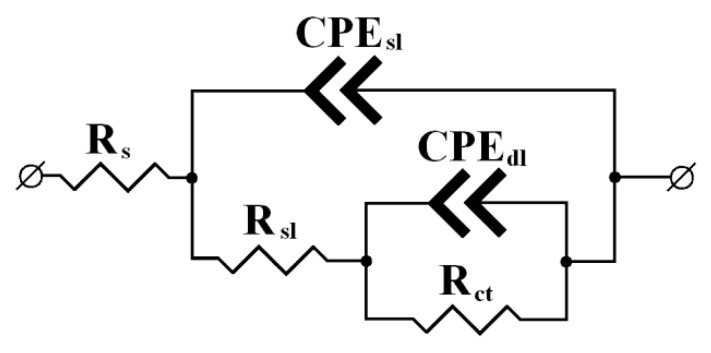
The equivalent circuit used to calculate the impedance parameters.

Electrochemical impedance spectra were recorded using a potentiostat of the same brand and a frequency response analyzer (FRA) also manufactured in the Russian Federation. The experiments were performed in a cell, on electrodes, and under conditions similar to those described above. The frequency was varied from 0.1 to 10^5^ Hz. Electrochemical impedance parameters were calculated using the equivalent circuit that is widely used to describe the impedance spectra of magnesium alloys [44] (Figure 1).

In this case, *R_s_* is the resistance of the bulk electrolyte between the auxiliary and working electrodes, which does not affect the electrode processes and depends on the medium conductivity and cell geometry; *R_sl_* is the resistance of the surface layers, i.e., oxide-hydroxide layers (OHL) and adsorption layers; *R_ct_* is the polarization resistance that characterizes the electrochemical kinetics of the corrosion process; *CPE_sl_* is the constant phase element that characterizes the capacitance of the surface oxide-hydroxide layers and/or adsorption film; and *CPE_dl_* is the capacitance of the double electric layer in the film defects.

The results were processed with Dummy Circuits Solver software, version 2.1, to determine the equivalent circuit parameters. The fit between the experimental and calculated data was no worse than 98%.

The degree of magnesium electrode protection was calculated by the following formula:

*Z* = (*R_inh_* − *R_bg_*)/*R_inh_* × 100% (1)

In this equation, *R_bg_* and *R_inh_* are the total resistance of “metal-electrolyte” interphase interaction, including *R_ct_* and *R_sl_* after thermal treatment of the electrode in the absence and in the presence of CBTA, respectively.

### 2.6. X-ray Photoelectron Spectroscopy

The thickness of the films (*d*) formed on the metals during CT was estimated by analyzing the intensities of X-ray photoelectron spectra (XPS). The spectra of C1s, O1s, N1s and Mg2p electrons were recorded on an Omicron ESCA+ instrument (Germany) with a Mg anode as the radiation source. The pressure in the analyzer chamber was no higher than 10^−9^ Torr. The analyzer energy was 20 eV. The positions of lines of the elements in the surface layer were standardized versus the C1s peak, whose energy was taken to be 285.0 eV. The peak intensities were obtained after subtracting the background by the Shirley method [45]. The thicknesses of the layers formed on the surface were calculated using the MultiQuant 7.70 program [46], taking the photoionization cross-sections of atoms [47] and the electron mean free paths [48] into account.

### 2.7. Scanning Electron Microscopy

The morphology of the protective coatings formed was studied using a Quanta 650 FEG field cathode scanning electron microscope (FEI, Netherlands) under high vacuum, at an accelerating voltage of 2 kV. For this purpose, the prepared samples were mounted on an aluminum holder by double-sided conductive carbon tape and placed into the device chamber.

All the values provided in the article were obtained by averaging 10–15 independent experiments.

## 3. Results and Discussion

### 3.1. Corrosion Tests

**Figure 2:**
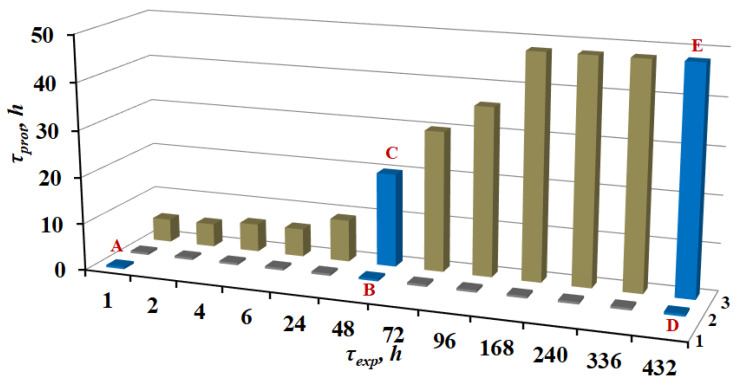
Effect of *τ*_exp_ on the *τ*_prot_ of magnesium alloy samples treated under different conditions. Row 1—neither TT nor CT; row 2—after TT; row 3—after CT. The results obtained for the samples prepared in modes **A**, **B**, **C**, **D**, and **E** are highlighted in color.

The results of corrosion tests are shown in Figure 2.

For the samples that did not undergo either TT or CT, the first sign of corrosion damage in the form of separate dark spots appeared after 0.5 h of exposure to corrosive conditions. Subsequently, the spots grew, until 100% surface damage was observed in the experiments 3–5 h long.

One-hour thermal treatment of samples at 150 °C without a CIN did not affect the corrosion behavior of the alloy.

For the samples treated with hot CBTA vapor for *τ_exp_* = 1 h, the time before the first sign of corrosion damage was detected increased 10-fold (to 5 h). The nature of corrosion damage changed in comparison with the background samples. Corrosion occurred as separate points, the number of which increased with time. An increase in τ_exp_ resulted in an increase in the protection efficiency of the treated samples. The maximum value of *τ_prot_* (48 h) was achieved at *τ_exp_* = 168 h. A further increase in *τ_exp_* up to 432 h failed to increase the corrosion resistance of the alloy.

Comparison of the samples prepared in modes **A**, **B** and **D** shows that TT by itself does not affect the corrosion behavior of the magnesium alloy, regardless of *τ_exp_*. Chamber treatment of the samples slows down the initiation of alloy corrosion (*cf*. the samples pretreated in modes **B** and **C**). Moreover, the exposure of samples in air after CT (preparation modes **C** and **E**) improves the corrosion resistance of the metal.

Thus, direct corrosion experiments confirm the conclusion [32] about the structuring ability of surface films formed on a magnesium alloy in hot CBTA vapors upon exposure of the samples in air after CT. These processes are accompanied by an increase in the corrosion resistance of the alloy, up to 10-fold with respect to the samples not exposed to air after CT, and up to 100-fold with respect to the original magnesium samples.

### 3.2. Voltammetry

**Figure 3:**
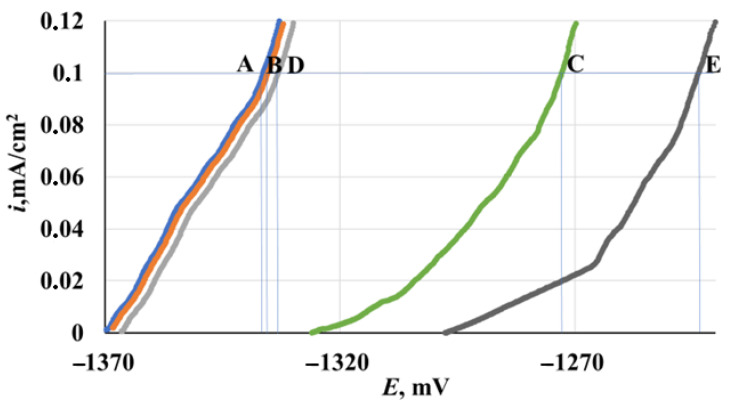
Anodic polarization curves of electrodes treated in modes **A**, **B**, **C**, **D** and **E**.

The polarization curves of the samples prepared in modes **A**, **B**, **C**, **D** and **E** are shown in Figure 3.

Irrespective of the conditions for the preparation of the magnesium alloy, the polarization curves show two nearly linear regions of *i* increase. The first one has a relatively small slope, whereas the second one is characterized by a sharp *i* increase. It is hardly possible to reliably attribute the former region to the alloy dissolution from the passive state and the latter one to local dissolution, as it is often the case for passive metals. The *i* values already reach considerable values in the first region of the anodic polarization curves. In this case, the surface of the metal removed from the electrolyte is heavily etched and is covered with a dark gray deposit. This prevents a visual identification of local metal dissolution sites in the second linear region of the polarization curves. It should be noted that small current oscillations are present throughout the entire length of the anodic polarization curves. It may be assumed that they result from hydrogen bubbles on the electrode surface that are formed due to the negative difference effect and/or chemical dissolution of the magnesium alloy.

The polarization curves of magnesium prepared in modes **A**, **B**, and **D** are quite similar. Their characteristic values are nearly the same (*E*_st_ and *E_i_* are −1.37 and −1.34 V, respectively).

The character of the anodic polarization curves on electrodes treated in CBTA vapors (preparation modes **C** and **E**) changed somewhat. The initial region had a smaller slope with respect to the abscissa axis. The polarization curves themselves shifted to the anodic region due to the inhibition of the anodic process. The *i* values decreased abruptly in the entire potential range studied. Moreover, the preparation mode **E** resulted in much lower *i* values compared to mode **C**. With an increase in *τ_exp_*, ennoblement of both *E*_st_ and *E_i_* was observed.

Thus, comparison of the voltam metric plots of electrodes prepared in modes **A**, **B** and **D** shows that TT of samples does not affect the anodic behavior of the magnesium alloy, regardless of *τ_exp_*. This fact agrees with the results of the corrosion tests reported above. Chamber treatment of electrodes slows down the anodic process (*cf*. the samples pretreated in modes **B** and **C**). It is noteworthy that exposure of the samples in air after CT (preparation modes **C** and **E**) causes additional inhibition of the anodic process.

### 3.3. Electrochemical Impedance Spectroscopy

**Figure 4:**
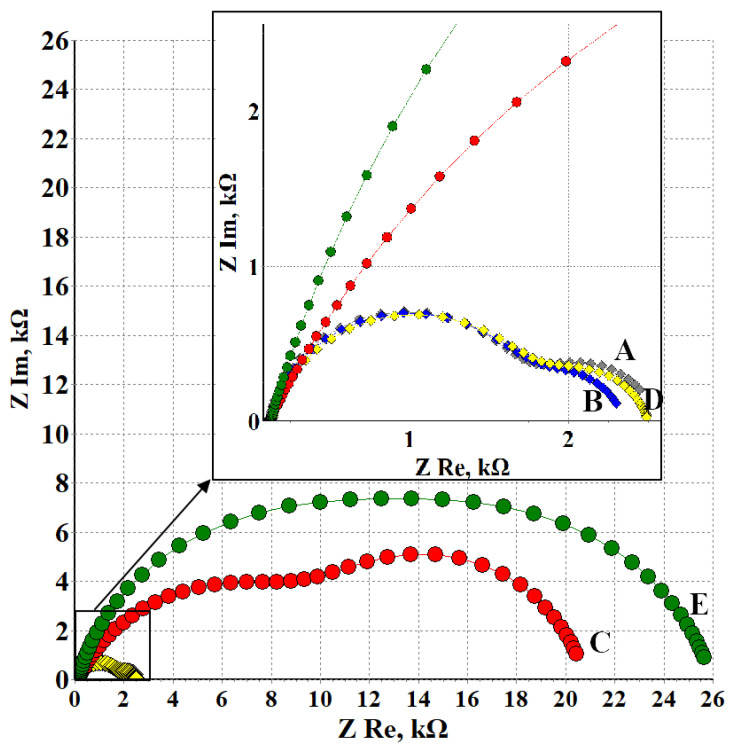
Nyquist plots of electrodes treated in modes **A**, **B**, **C**, **D** and **E**.

Additional information on the mechanisms and kinetics of the structuring processes in surface films on the magnesium alloy is provided by electrochemical impedance spectroscopy. The Nyquist plots of electrodes treated in modes **A**, **B**, **C**, **D** and **E** are shown in Figure 4.

All the hodographs presented here are characterized by two semicircles, which allows them to be described by a general equivalent circuit with two time constants (Figure 1).

In the scope of the selected model, the high-frequency (small) semicircle on the hodograph corresponds to the time constant that is mainly associated with the *R_sl_*/*CPE_sl_* circuit, i.e., it depends on the conductivity of the surface layer. The low-frequency (large) hodograph arc is associated with the kinetics of the Faraday reaction on the metal, which actually determines its corrosion behavior. The parameters of this semicircle are described by the *R_ct_*/*CPE_dl_* circuit as part of the overall equivalent circuit.

One can observe from Figure 4 that the radii of the arcs in the hodographs of the samples treated with CBTA vapors (modes **C** and **E**) are significantly larger than those of the samples before the TT (**A**) or after the TT without an inhibitor (**B**,**D**). This qualitatively indicates that CBTA manifests a strong inhibitive effect.

Analysis of the calculated equivalent circuit parameters (Table 2) shows that heat treatment of a sample followed by exposure to air for 48 h (mode **B**) or 432 h (conditions **D**) increased *R_sl_* insignificantly. This effect may be due to some growth of the OHL. The values of *CPE_sl_* and *n_sl_* that are close under the conditions used do not contradict this assumption. The *R_ct_* values obtained after TT and exposure of electrodes in air for 48 h slightly decreased with respect to the original electrode. This is probably due to the formation of defects in the surface OHL during TT. This assumption is confirmed by a decrease in *CPE_dl_* compared to the background value (**A**), which can be interpreted as an increase in the electrochemically active surface area. *R_ct_* slightly increased with an increase in the exposure time, while *CPE_dl_* did not change. Most likely, the changes in *R_ct_* are due to the growth and modification of surface layers upon exposure to air. This can also explain the insignificant increase in the double layer inhomogeneity (the decrease in *n_dl_*) upon exposure of the electrodes to air. The calculated Z values indicate a certain acceleration of corrosion processes on the magnesium alloy after TT and exposure to air. This does not contradict the results of corrosion experiments, where the samples were examined every hour. It is impossible to detect such a small corrosion acceleration in such inspections.

CT of the magnesium alloy in CBTA vapors followed by its exposure to air for 48 h (mode **C**) resulted in a more than 7-fold increase in the resistance of surface layers, *R_sl_*. It increased symbatically to *τ_exp_* to reach almost 17,000 Ohm after 432 h of exposure to air (mode **E**). The capacitance of the surface layers (modulus A of the *CPE_sl_* element) decreased significantly upon prolonged exposure of the electrodes to air. This indicates that changes occurred in the structure and dielectric properties of the surface layers. The phase coefficient *n_sl_* increased with an increase in *τ_exp_* to 432 h in comparison with treatment mode **C**, which indicates that the heterogeneity of the surface layers decreased.

With regard to the circuit elements responsible for the low-frequency process (corrosion kinetics), the charge transfer resistance after treatment in CBTA vapors and exposure to air for 48 h increased more than 11-fold compared to the background value. At the same time, the double layer capacitance decreased 50-fold. An increase in *τ_exp_* to 432 h insignificantly increased *R_ct_* and decreased the double layer capacitance. The *n_dl_* value also decreased only slightly. The range of calculated *n_dl_* values (0.81–0.9) allows us to state that *CPE_dl_* is almost an ideal capacitor, in which Faraday processes occur on a homogeneous surface and are not complicated by diffusion. The calculated Z values indicate significant inhibition of corrosion processes on the magnesium alloy upon its CT followed by exposure to air, which is consistent with the results of the corrosion and voltam metric experiments.

The results of the simulating experimental data based on the equivalent circuit make it possible to make quantitative estimates of the contribution of various mechanisms that provide the inhibitive effect of CBTA.

Two principal mechanisms of action of adsorption-type corrosion inhibitors are the blocking and activation mechanisms [31]. In the former case, an inhibitor is adsorbed and blocks a fraction of the metal surface, thus reducing the corrosion rate, but does not affect the kinetics of electrode processes on the remaining unblocked surface. In contrast, the activation mechanism implies that corrosion inhibition is due to a change in the activation energy, and hence the kinetics of the corrosion processes. Both mechanisms usually operate simultaneously, but their contributions to the inhibitory effect may differ.

In accordance with [49], the *R_sl_* value in the equivalent circuit can serve as a criterion for estimating the blocking effect of the inhibitor. The coefficient of corrosion inhibition due to surface blocking (*γ_sl_*) is the ratio of resistance *R_sl_* of the sample after CT and after TT without a CIN.

*γ_sl_ = R_sl_^CT^/R_sl_^TT^* (2)

Using a similar approach, the *R_ct_* value can be used to estimate the effect of a CIN on the Faraday corrosion process. Hence, the coefficient of electrochemical reaction inhibition by a CIN (*γ_ct_*) can be determined as the ratio of the charge transfer resistance *R_ct_* for inhibited and non-inhibited samples.

*γ_ct_ = R_ct_^CT^/R_ct_^TT^* (3)

The calculated values of *γ_sl_* and *γ_ct_* are presented in Table 3.

The analysis of γ_sl_ and γ_ct_ values implies a mixed blocking-activation mechanism of CBTA action. After preparation of the samples in both modes (**C** and **D**), the inequality *γ_sl_* < *γ_ct_* is observed, which indicates that the activation inhibition mechanism predominates. However, mode **D** results in a smaller *γ_ct_*/*γ_sl_* ratio. This allows us to conclude that the contribution of the blocking effect to the protective effect of the inhibitor grows upon exposure of the electrodes to air after CT.

### 3.4. X-ray Photoelectron Spectroscopy

**Figure 5:**
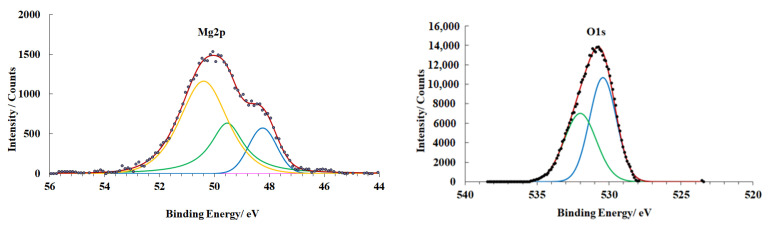
XPS spectra of magnesium alloy samples (preparation mode **A**).

The surface of the magnesium alloy in the original state (preparation mode **A**) contains an OHL. Its XPS spectra are shown in Figure 5.

The peak with an energy of 48.7 eV in the Mg2p spectrum corresponds to metallic magnesium, while the peaks at 49.5 eV and 50.6 eV correspond to magnesium hydroxide and oxide, respectively. In the O1s spectrum, the peak at 530.5 eV corresponds to magnesium oxide, while the peak at 531.9 eV to the hydroxide.

The thickness of the oxide layer directly adjacent to the magnesium surface is 2.5 nm, while the thickness of the hydroxide film located above it is 1.8 nm (Table 4).

**Figure 6:**
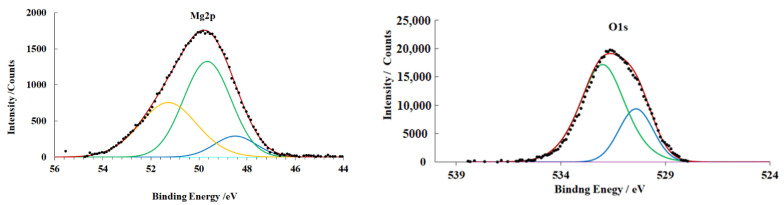
XPS spectra of magnesium alloy samples (preparation mode **B**).

The same peaks are visible in the spectra of the samples prepared in mode **B** (Figure 6), but their intensity changes. A decrease in the peaks of metallic magnesium and MgO in comparison with the samples prepared in mode **A** is observed. This peak intensity change is due to the growth of the hydroxide layer.

**Figure 7:**
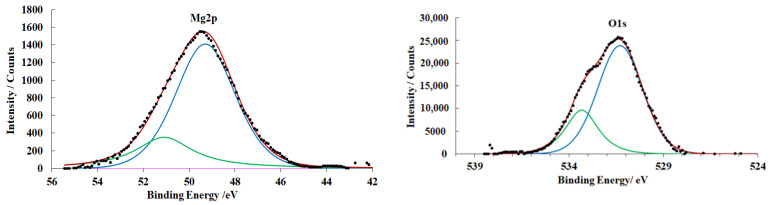
XPS spectra of magnesium alloy samples (preparation mode **D**).

As the samples heat-treated without an inhibitor are kept in air (preparation mode **D**), magnesium oxidizes with the thickening of the hydroxide layer, as it follows the increase in the integral intensity of the hydroxide peak in the Mg2p and O1s spectra (Figure 7). The total thickness of the OHL increases by almost 3 nm after 18 days of exposure to air.

It is important that a peak with an energy of 533.6 eV that belongs to water molecules appears in the O1s spectrum. The presence of water molecules in the OHL is apparently due to its porous structure and atmospheric humidity.

**Figure 8:**
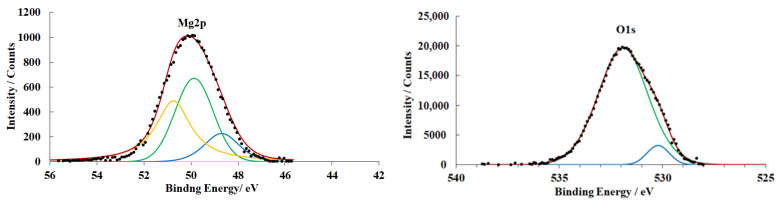

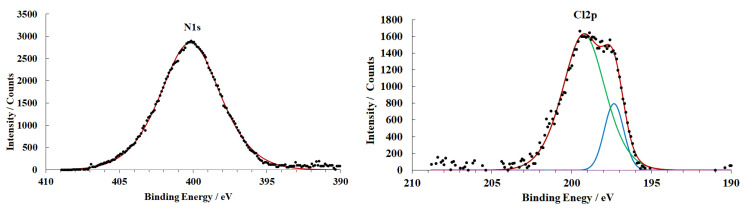
XPS spectra of magnesium alloy samples (preparation mode **C**).

After CT in CBTA vapors (preparation mode **C**), a chemisorption layer is formed on the magnesium surface. It is identified by the appearance of nitrogen and chlorine peaks in the spectra (Figure 8).

The N1s XPS spectrum exhibits a singlet peak at 400.1 eV that belongs to the nitrogen atoms of the azole ring. According to literature data [50], the electron density is distributed uniformly on the nitrogen atoms of the azole ring. Therefore, a single symmetric peak is observed in the spectra. The position of the maximum in the N1s spectrum indicates that the interaction is of chemisorption nature [51].

The Cl2p spectrum contains a doublet peak that belongs to the Cl2p^1/2^ and Cl^3/2^ photoelectrons of chemisorbed CBTA molecules.

The presence of a chemisorbed CBTA layer hinders magnesium oxidation. The total thickness of the OHL is 1.3 nm smaller than that in the case of the magnesium alloy treated in mode **B**.

**Figure 9:**
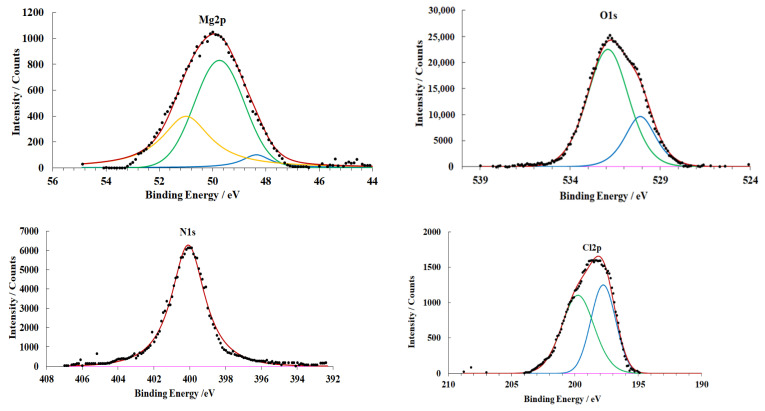
XPS spectra of magnesium alloy samples (preparation mode **E**).

After CT, followed by exposure of the samples to air for 18 days (preparation mode **E**), the XPS spectra (Figure 9) show changes associated with the growth of the hydroxide layer. However, they are smaller than on the samples prepared in mode **D**. The OHL on the metal is thinner by 3.3 nm. The presence of a metallic magnesium peak indicates that the growth of the hydroxide layer occurs in areas not protected by the inhibitor. In this case, the Cl2p spectrum changes only slightly after exposure of the samples to air. This indicates that CBTA does not undergo desorption.

To summarize the results of the XPS study, it should be noted that the comparison of the XPS spectra of the samples prepared in modes **A** and **B** indicates that TT of magnesium results in its oxidation and transition of a fraction of the oxide to the hydroxide. These processes result in the thickening of the OHL upon TT without an inhibitor.

Exposure of the heat-treated magnesium alloy in air (cf. the spectra of samples treated in modes **B** and **D**) is accompanied by oxidation of magnesium and hydroxylation of the oxide. These processes result in the thickening of the OHL where water appears. The presence of the latter may apparently be explained by the porous structure of the surface layer.

CT in hot CBTA vapors results in its chemisorption on the alloy surface (cf. the samples treated in modes **B** and **C**). CT also causes the thickening of the magnesium oxide film, although it is less pronounced than in the case of TT. Hydroxylation of the oxide is inhibited by chemisorption of the inhibitor. The sum of the processes that occur during CT result in the thinning of the OHL, in comparison with the metal heat-treated without an inhibitor.

Exposure of the samples to air after CT is accompanied by magnesium oxidation and hydroxide film growth (treatment modes **C** and **E**).The integral thicknesses of the surface films increase upon exposure to air. The data we obtained do not allow us to state unambiguously whether a uniform thickening of the OHL occurs or if the process is of “islet” nature.

The version of the islet growth of OHL is confirmed by electron microscopy data.

### 3.5. Scanning Electron Microscopy

**Figure 10:**
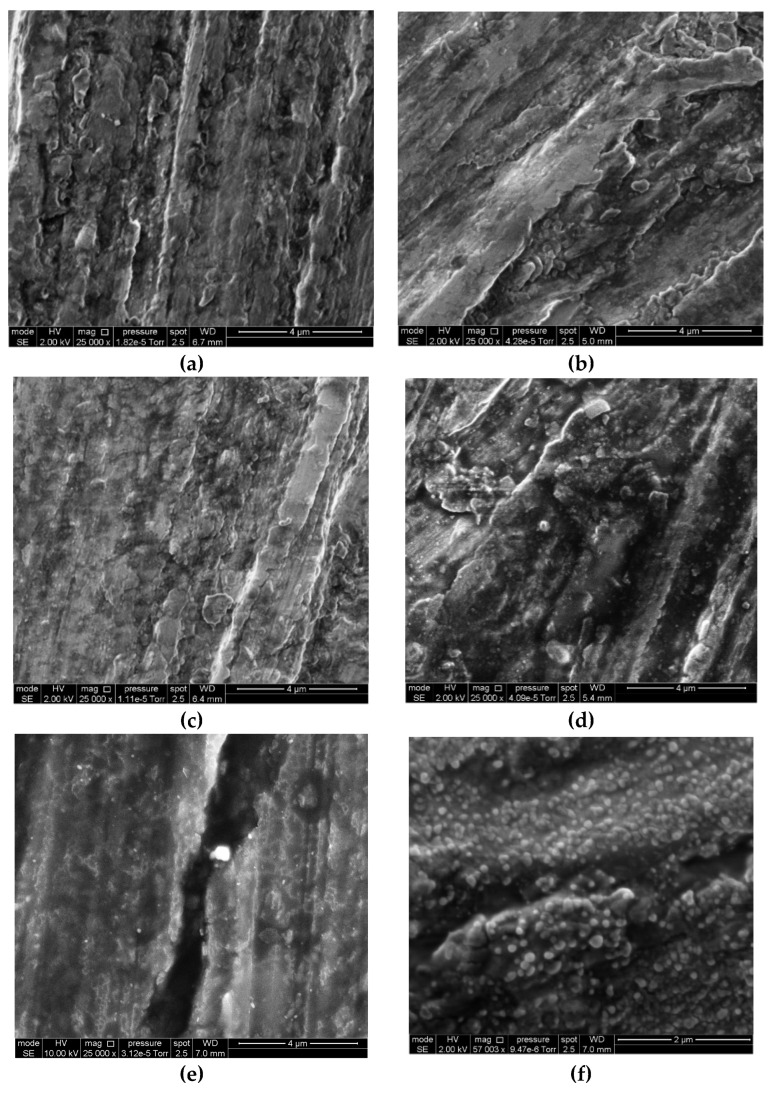
Micrographs of magnesium alloy samples. **a**—original state, **b**—original state, *τ*_exp_ = 720 h; **c**—after TT, *τ_exp_* = 24 h; **d**—after TT, *τ*_exp_ = 720 h; **e**—after CT, *τ*_exp_ = 24 h; **f**—after CT, *τ*_exp_ = 720 h.

Figure 10 shows typical micrographs of the original and modified magnesium alloy samples. The conditions under which the samples shown in the micrographs were prepared did not fully match the conditions of modes **A**, **B**, **C**, **D** and **E**. Nevertheless, the analysis of these images supplements the above studies of the processes that occur on a magnesium alloy during exposure to air after CT.

The surface structure of the magnesium alloy in the original state, the structure after TT followed by exposure to air for 24 or 720 h, and the structure in 24 h after CT are very similar. However, prolonged exposure of the samples after CT results in the formation of single rounded lumps 70–200 nm large on the surface. We have reason to believe that the integral thickening of the OHL inferred from the XPS spectra is due to its islet-like growth.

It should be noted that the SEM studies presented above do not explain the data obtained by other methods but do not contradict them either. So far, we have only stated the experimental fact that oxide-hydroxide layers grow in the islet mode during exposure of magnesium to air after the CT in CBTA vapors. We also observed the growth of such islets on some other metals under similar conditions. Now, we are beginning a study that we hope will clarify the role of the islet growth of oxide-hydroxide layers in increasing the corrosion resistance of metals following chamber treatment.

Thus, analysis of the results of corrosion, electrochemical and physical investigation methods allows us to draw the general picture of the processes that occur upon chamber treatment of the magnesium alloy by CBTA, which is presented in the conclusions below.

## 4. Conclusions

1. A fairly uniform oxide-hydroxide layer with a thickness of about 4.3 nm exists on the original surface of the magnesium alloy.
2. Heat treatment results in the growth of the oxide-hydroxide layer to 6.5 nm. It is not accompanied by changes in the corrosion-electrochemical behavior of the magnesium alloy or in the surface image in the micrographs.
3. Exposure of the samples to air after heat treatment is accompanied by additional oxidation of magnesium and hydroxylation of the oxide. These processes result in the thickening of the oxide-hydroxide layer to 9.4 nm. Moreover, water appears in the layer. The thickening of the film is uniform and does not result in changes in the corrosion-electrochemical behavior of the magnesium alloy or to changes in the surface appearance in the micrographs.
4. Chamber treatment of the magnesium alloy is accompanied by CBTA adsorption. The oxide-hydroxide layer grows to 5.2 nm. This growth is somewhat inhibited by CBTA adsorption. CBTA adsorption slows down corrosion initiation about 10-fold and inhibits magnesium dissolution, due to inhibition of the anodic process. Inhibition occurs by a mixed blocking-activation mechanism with predominance of the latter. The appearance of the surface in the micrographs does not change upon chamber treatment of the magnesium alloy.
5. Prolonged exposure of the samples after the chamber treatment is accompanied by additional oxidation of magnesium and hydroxylation of the oxide. The integral thickness of the oxide-hydroxide layer increases to 6.1 nm. However, the oxide-hydroxide layer does not grow on the entire surface, but as separate islets, whose sizes may be up to 70–200 nm. No noticeable desorption of CBTA occurs upon exposure to air. The above processes are accompanied by additional (~100-fold) inhibition of corrosion initiation and anodic dissolution of magnesium. Inhibition occurs by a mixed blocking-activation mechanism with predominance of the latter; however, the contribution of the blocking mechanism increases with exposure to air.

## Figures and Tables

**Table 1:** Composition of MA8 alloy in mass% [43].

Fe	Si	Mn	Ni	Ce	Al	Cu	Be	Mg	Zn	Impurities
<0.05	<0.1	1.3–2.2	<0.007	0.15–0.35	<0.1	<0.05	<0.002	96.84–98.55	<0.3	0.3

**Table 2:** Impedance parameters calculated for electrodes treated in modes **A**, **B**, **C**, **D** and **E**.

Preparation Modes	*R_ct_* Ohm∙cm^2^	*CPE_dl_* (S s^n^/cm^2^)	*n_dl_*	*R_sl_* Ohm∙cm^2^	*CPE_sl_* (S s^n^/cm^2^)	*n_sl_*	*R_r_* Ohm∙cm^2^	Z, %
**A**	746	5.9 × 10^−4^	0.85	1693	8.7 × 10^−6^	0.88	119	
**B**	499	5.8 × 10^−4^	0.86	1724	9.7 × 10^−6^	0.86	123	−9.71
**C**	8704	1.17 × 10^−5^	0.90	12,029	1.78 × 10^−6^	0.77	118	88.24
**D**	615	5.8 × 10^−4^	0.83	1755	9.6 × 10^−6^	0.84	131	−2.91
**E**	8991	9.2 × 10^−6^	0.81	16,832	1.01 × 10^−6^	0.86	123	90.55

**Table 3:** Inhibition coefficients for samples prepared in modes **C** and **E**.

Preparation Conditions	*γ_sl_*	*γ_ct_*	*γ_ct_*/*γ_sl_*
**C**	7.0	17.4	2.43
**E**	9.8	18.0	1.83

**Table 4:** Integral intensity of peaks in Mg2p XPS spectra and total thickness of the OHL upon sample preparation in different modes.

Sample Preparation Mode	Mg, %	MgO, %	MgOxOHy, %	OHL Thickness, nm
**A**	30.4	46.2	23.4	4.3
**B**	6.2	28.6	65.2	6.5
**C**	16.1	34.3	49.6	5.2
**D**	-	21.8	78.2	9.4
**E**	11.4	32.4	56.2	6.1

## Data Availability

Not applicable.
